# *Laurus nobilis* L. Essential Oil-Loaded PLGA as a Nanoformulation Candidate for Cancer Treatment

**DOI:** 10.3390/molecules27061899

**Published:** 2022-03-15

**Authors:** Esin Ercin, Serda Kecel-Gunduz, Bahar Gok, Tugba Aydin, Yasemin Budama-Kilinc, Murat Kartal

**Affiliations:** 1 Department of Pharmacognosy and Natural Product Chemistry, Institute of Health Sciences, Bezmialem Vakıf University, Istanbul 34093, Turkey; esin_ercin@yahoo.com (E.E.); tugba.aydin@istinye.edu.tr (T.A.); 2 Department of Physics, Faculty of Science, Istanbul University, Istanbul 34134, Turkey; skecel@istanbul.edu.tr; 3 Department of Bioengineering, Graduate School of Natural and Applied Science, Yildiz Technical University, Istanbul 34220, Turkey; bahar.991@hotmail.com; 4 Department of Pharmacognosy, Faculty of Pharmacy, Istinye University, Istanbul 34010, Turkey; 5 Department of Bioengineering, Faculty of Chemical and Metallurgical Engineering, Yildiz Technical University, Istanbul 34220, Turkey; 6 Department of Pharmacognosy, Faculty of Pharmacy, Bezmialem Vakif University, Istanbul 34093, Turkey; mkartal@bezmialem.edu.tr

**Keywords:** laurel essential oil, PLGA, controlled release system, nanoparticle, DNA binding

## Abstract

The aim of this study was to obtain essential oil (LNEO) from the *Laurus nobilis* L. plant, and to prepare LNEO-loaded poly lactic-co-glycolic acid (PLGA) nanoparticles (NPs) as an approach in cancer treatment. The components of the obtained LNEO were analyzed using GC-MS. The LNEO-NPs were synthesized by the single-emulsion method. The LNEO-NPs were characterized using UV-Vis spectrometry, Dynamic Light Scattering (DLS), Scanning Electron Microscopy (SEM), and a DNA binding assay, which was performed via the UV-Vis titration method. According to the results, the LNEO-NPs had a 211.4 ± 4.031 nm average particle size, 0.068 ± 0.016 PdI, and −7.87 ± 1.15 mV zeta potential. The encapsulation efficiency and loading capacity were calculated as 59.25% and 25.65%, respectively, and the in vitro drug release study showed an LNEO release of 93.97 ± 3.78% over the 72 h period. Moreover, the LNEO was intercalatively bound to CT-DNA. In addition, the mechanism of action of LNEO on a dual PI3K/mTOR inhibitor was predicted, and its antiproliferative activity and mechanism were determined using molecular docking analysis. It was concluded that LNEO-loaded PLGA NPs may be used for cancer treatment as a novel phytotherapeutic agent-based controlled-release system.

## 1. Introduction

According to the World Health Organization (WHO), cancer is the second leading cause of death worldwide, accounting for around 10 million deaths annually. Globally, one in six deaths are associated with cancer. It is estimated that cancer-related deaths will increase by 70% in 2027 [1]. Cancer-causing infections, such as hepatitis and human papilloma virus (HPV), are responsible for approximately 30% of cancer cases in low- and middle-income countries [2]. More than 90% of high-income countries have comprehensive treatment, while less than 15% of low-income countries have this opportunity [3].

Traditional cancer treatment includes different interventions such as chemotherapy, surgery and radiation therapy; however, these treatment modalities also damage the adjacent healthy cells [4]. New anti-proliferative drugs are used for cancer treatment, but they come with severe adverse effects, toxicity and have high costs. Therefore, cost-effective and biocompatible therapeutic applications are needed [5]. New natural products will play an important role in this period. Many of the antitumor agents currently used in clinical practice are of natural origin. Among the great diversity of phytochemicals, essential oils have attracted great attention due to their diverse bioactivity. It is known that many essential oils show strong antioxidant activity, and are also antimutagenic and anticarcinogenic. They prevent the proliferation of tumor cells due to their antiproliferative effects. The literature supports the supposition that essential oils have potential therapeutic activities in the prevention of cancer [6].

A few studies with laurel essential oil have shown that it has antiproliferative and cytotoxic activities on cancer cells. It also has antifungal, antimicrobial, nematicidal, antibacterial, antioxidant, insecticidal, anticonvulsant and acaricidal activities [7]. Laurel essential oil is usually obtained via hydrodistillation or steam distillation. Bay leaf and flower essential oils contain 1,8-cineol, α-terpinyl acetate and methyleugenol as their main components [7,8]. Turkish *Pharmacopoeia* includes bay leaf and bay leaf essential oil as national monographs. Bay leaf essential oil contains at least 48% 1,8-cineole and 7% α-terpinyl acetate. According to these monographs, it has a clear, yellow, characteristic aroma, spicy-smelling and camphor-flavored liquid [9]. Due to their structural relationship within the same chemical group, essential oil components are known to easily convert from one to another by oxidation, isomerization, cyclization, or dehydrogenation reactions, which are induced either enzymatically or chemically. For the stability evaluation of essential oils, it needs to be kept in mind that the chemical composition may already vary in the starting material, being influenced by the plant’s health, growth stage, habitat (including climate), edaphic factors, and the harvest time [10]. Encapsulation is a process in which small solid particles, liquid components or gaseous materials are covered with or trapped in another inert shell material, isolating and protecting the core material from environmental factors. Encapsulation has also been shown to improve the antibacterial activity of several antibiotics. However, due to its high volatility and sensitivity to environmental effects, laurel essential oil especially needs encapsulation in order to facilitate its use and increase its bioavailability. Through encapsulation, the stability of laurel essential oil can be increased and its pungent odor can be masked [11,12].

Recently, encapsulation with nanocarrier systems has emerged as a promising intervention in cancer therapy. Nanotechnology is focused on the reduction of toxicity and the improvement of the bioavailability of drugs in target tumor cells. Nanocarrier-loaded therapeutic drug delivery methods have shown promising potential in the treatment of cancer, as they target the control of the growth of tumor cells [5].

Biodegradable polymeric nanoparticles are widely used as delivery systems of active ingredients. Polymeric nanoparticles protect active compounds from degradation [13]. Synthetic polymers have the advantage of higher purity and reproducibility than natural polymers. PLGA has been approved by the US Food and Drug Administration for 14 pharmaceutical and biomedical applications [14]. PLGA polymer is of great interest to the biomedical field due to its biocompatibility, biodegradability and controlled distribution properties [15,16].

In this study, bay leaves were collected from the Aegean Agricultural Research Institute (Izmir, Turkey), and laurel leaf essential oil was obtained by the hydrodistillation method according to the European Pharmacopoeia. The obtained laurel leaf essential oil was analyzed by GC-FID/MS. The average particle size, polydispersity index value (PdI) and zeta potential value were obtained according to dynamic light scattering principles, and the morphology of the nanoparticles was confirmed by SEM. The encapsulation efficiency, loading capacity and release profile were determined using a UV-Vis spectrophotometer.

The molecular docking approach is used to characterize the behavior of small molecules in the binding site of target macromolecules such as proteins, enzymes, and DNA, etc., allowing for the prediction of basic biochemical processes by modeling the interactions between small molecules and macromolecules at the atomic level; therefore, it is of great importance in the evaluation process of potential new drugs. With the molecular docking analysis method, we aimed to reveal the inhibitory effect and antiproliferative properties of the most dominant components in laurel essential oil (LNEO) in relation to the PI3K/mTOR target receptor.

## 2. Results

### 2.1. LNEO Composition

Dry *Laurus nobilis* leaf yielded 1.98 ± 0.16 mL of essential oil/100 g on average after three rounds of hydrodistillation. A total of 11 compounds in laurel leaf essential oils were detected and quantified. Table 1 presents the constituents in terms of the retention indices’ order. The components of *Laurus nobilis* leaf essential oil were found to be 64.556% 1.8 cineole as the major compound, 10.099% sabinene, and 6.180% α-pinene, respectively.

### 2.2. Average Particle Size, PdI and Zeta Potential Analysis Results

**Figure 1:**
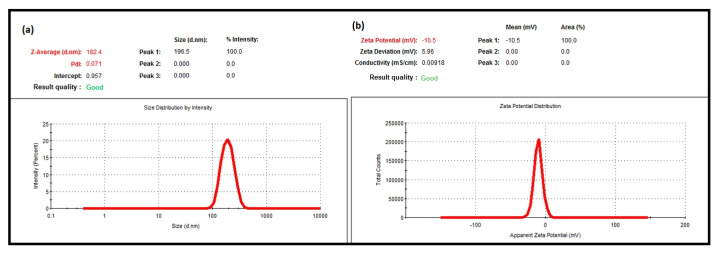
DLS analysis of the blank NPs: (**a**) average particle size, and (**b**) zeta potential graphs.

**Figure 2:**
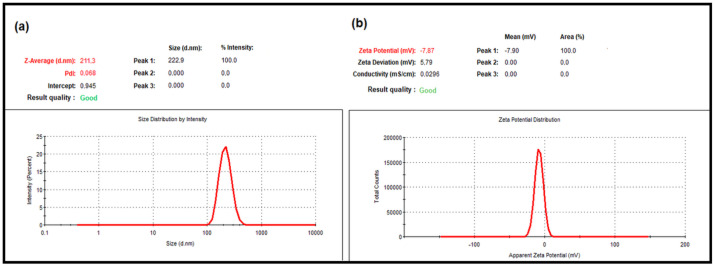
DLS analysis of the LNEO-NPs: (**a**) average particle size, and (**b**) zeta potential graphs.

The average particle size, PdI, and zeta potential of nanoparticles play an important role in the interaction of bioactive substances with the target tissue in drug delivery systems to produce a therapeutic effect [17,18,19]. DLS is one of the widely used methods to determine the average particle size, PdI, and zeta potential of synthesized nanoparticles [19,20,21]. In this study, the average particle size, PdI and zeta potential of the nanoparticles were determined using DLS. The DLS data showed that the mean particle size of the blank NPs was 182.4 ± 0.499 nm, and the PdI value was 0.071 ± 0.006 (Figure 1a). The zeta potential of the empty NPs was −10.5 ± 0.4 mV (Figure 1b). The mean particle size of the LNEO-NPs was 211.4 ± 4.031 nm, and their PdI was 0.068 ± 0.016 (Figure 2a). The zeta potential of the LNEO-NPs was −7.87 ± 1.15 mV (Figure 2b).

### 2.3. SEM Micrograph of the LNEO-NPs 

**Figure 3:**
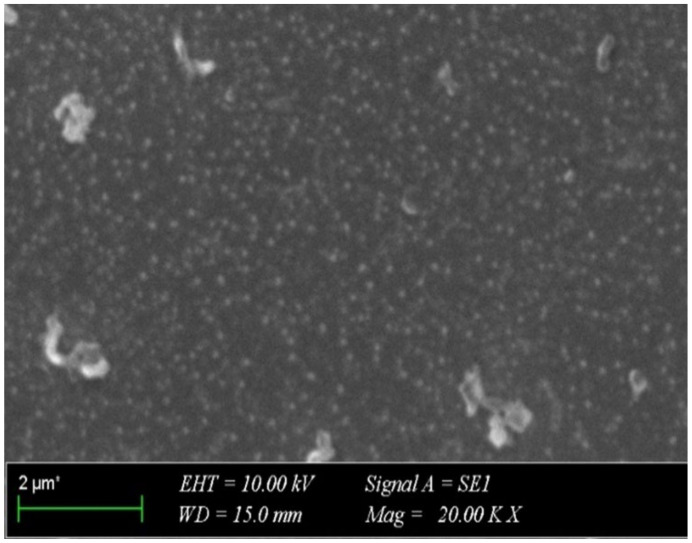
SEM image of the LNEO-NPs.

The morphology of the LNEO-NPs was determined using SEM. The SEM image (Figure 3) confirmed the synthesis of spherical LNEO-NPs [22], and showed that the LNEO-NPs were in a homogeneous size distribution.

### 2.4. Determination of the Encapsulation Efficiency and Loading Capacity

**Figure 4:**
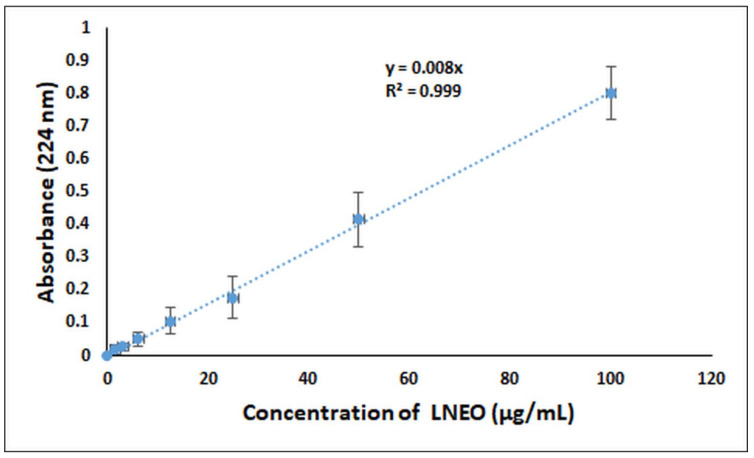
Standard curve of the LNEO.

The standard curve of the LNEO was prepared in order to determine the encapsulation efficiency and loading capacity of the LNEO-NPs (Figure 4). The encapsulation efficiency and loading capacity of the LNEO-loaded PLGA nanoparticles were calculated as 59.25% and 25.65%, respectively.

### 2.5. In Vitro Release Profile of the LNEO

**Figure 5:**
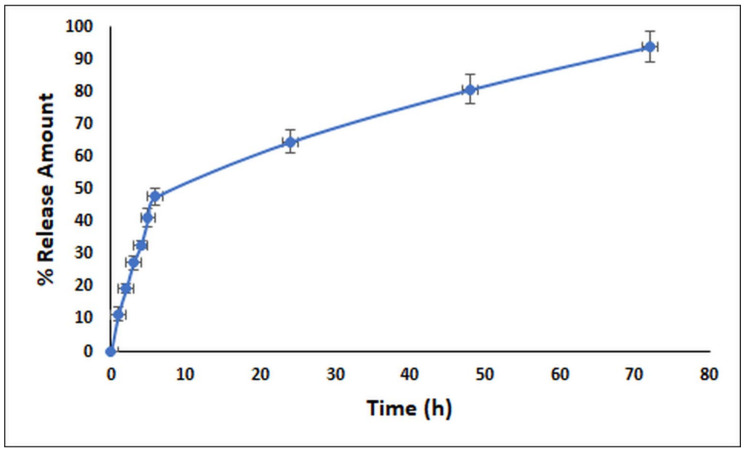
In vitro release profile of the LNEO (%).

The release profile of the LNEO-NP was evaluated as a function of time. Figure 5 shows the 72 h release results of the LNEO. The results showed that 47.76% ± 2.47% of the LNEO was released in the first six hours, and at the end of 72 h, 93.89% ± 4.93% of the LNEO had been released from the PLGA nanoparticles to the medium. 

### 2.6. DNA Binding

**Figure 6:**
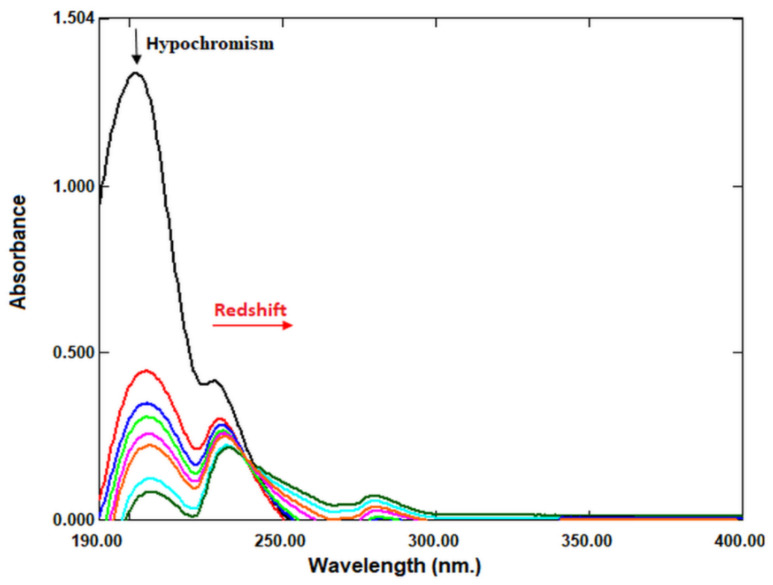
The absorption spectra of LNEO in the presence of increasing amounts of CT-DNA, and in the absence of CT-DNA (the black peak).

UV-Vis absorption spectrophotometry is a useful technique to investigate the interaction between anticancer drug molecules and DNA [23,24,25]. Therefore, we investigated the potential of LNEO as an anticancer drug molecule using UV-Vis absorption spectrophotometry. In the study, CT-DNA was used in the interaction of the LNEO with DNA. The absorption spectra of the LNEO in the presence and absence of CT-DNA are given in Figure 6. LNEO was found to exhibit a 93.80% hypochromic effect at the 201 nm wavelength, and 6 nm of bathochromic (red) shift. 

### 2.7. Molecular Docking and ADME Analysis Results

**Figure 7:**
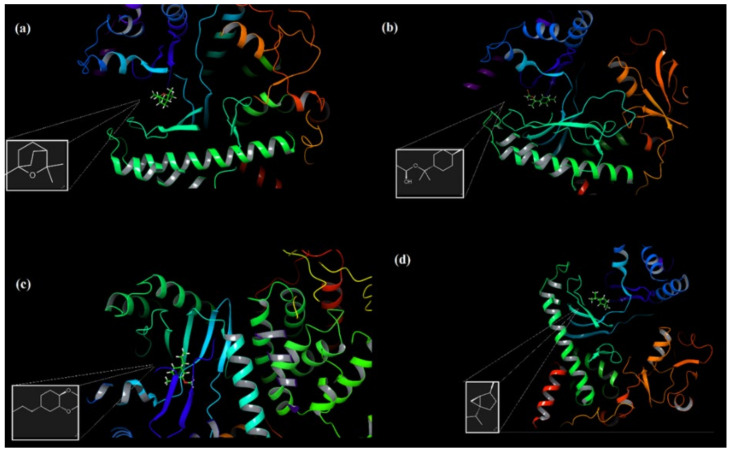
The docked pose of four major ingredients of LNEO: 1,8-cineole (**a**), α-terpinyl acetate (**b**), methyleugenol (**c**), and sabinene (**d**).

The inhibitory effects of the PI3K/mTOR target receptor by 1,8-cineole, α-terpinyl acetate, methyleugenol and sabinene molecules—which are the most dominant components in laurel essential oil (LNEO)—were analyzed, and their antiproliferative activity was evaluated by molecular docking analysis (Table 2). Table 2 also shows the ligand–receptor interactions within the target’s binding site, as well as the binding energy with each ligand. The docked poses of four major ingredients of LNEO are also shown in Figure 7 for 1,8-cineole, α-terpinyl acetate, methyleugenol and sabinene.

**Figure 8:**
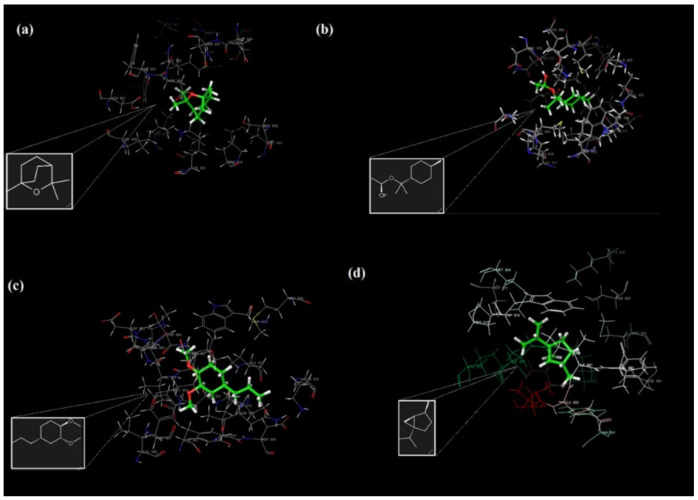
The active binding residues in the PI3K/mTOR target receptor (PDB 4FA6) and four docked major ingredients of LNEO: 1,8-cineole (**a**), α-terpinyl acetate (**b**), methyleugenol (**c**), and sabinene (**d**).

The active binding residues in the PI3K/mTOR target receptor (PDB 4FA6) were visualized for four major components in Figure 8 and Table 2.

**Figure 9:**
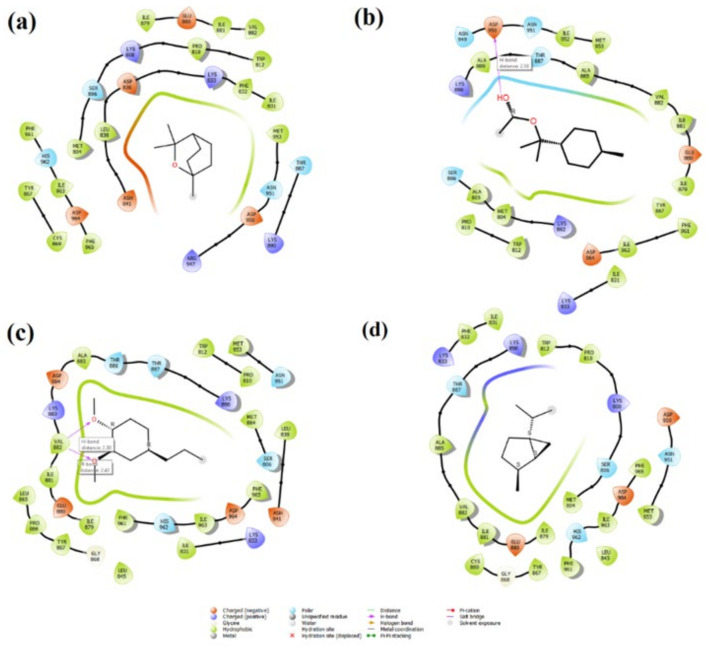
The 2D interactions of the major components of the LNEO (1,8-cineole (**a**), α-terpinyl acetate (**b**), methyleugenol (**c**) and sabinene (**d**)) with the PI3K/mTOR target receptor.

The amino acid Val 882, located in the binding pocket of 4FA6, is crucial for the inhibition of PI3Kα-mTOR; as can be seen from the interactions, all four major compounds are bound to the protein from this binding pocket, even by two separate hydrogen bonds (2.30Å and 2.43 Å) of methyleugenol with Val 882 (Figure 9c), which is the most stable binding component in this pocket, with a docking score energy of −5.97 kcal/mol. In addition, these four major compounds also showed hydrophobic interactions with Met 953, Ile 831, Ile 879 and Ile 963 by the co-crystallized ligand [26,27] interacting with the target receptor. α-Terpinyl acetate also has one hydrogen bonding interaction with Asp 950 in the PI3K/mTOR target receptor (Figure 9b).

**Figure 10:**
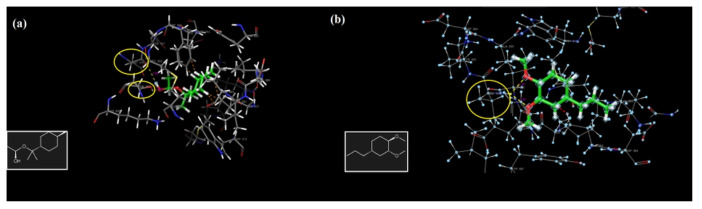
The hydrogen bonding interactions of methyleugenol with Val 882 (**a**), and α-terpinyl acetate with Asp 950 (**b**) in the PI3K/mTOR target receptor.

**Figure 11:**
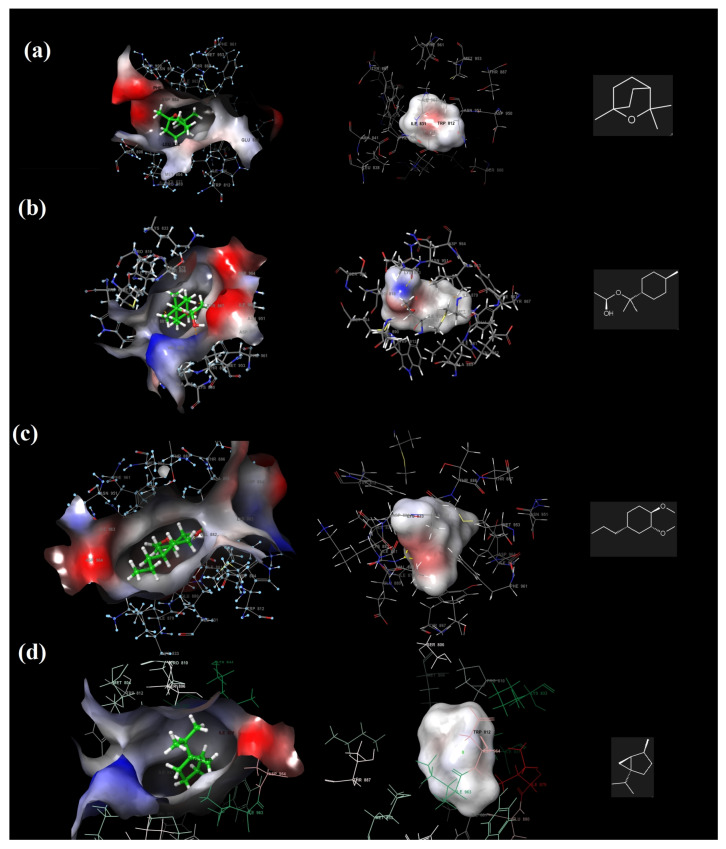
The electrostatic potentials of the binding pocket of the PI3K/mTOR target receptor and 1,8-cineole (**a**), α-terpinyl acetate (**b**), methyleugenol (**c**) and sabinene (**d**).

The hydrogen bonding interactions of methyleugenol with Val 882 (Figure 10a) and α-terpinyl acetate with Asp 950 in the PI3K/mTOR target receptor are illustrated in Figure 10. The electrostatic potentials of the binding pocket of the PI3K/mTOR target receptor and 1,8-cineole, α-terpinyl acetate, methyleugenol and sabinene molecules are also given in Figure 11, respectively.

Among the four major components, the 1,8-cineole molecule contains only negative electrostatic potential regions, which are shown in red; α-terpinyl acetate and methyleugenol both have negative electrostatic regions, shown in red, and positive potential regions shown in blue, while sabinene has no negative or positive regions (Table 3). For sabinene, the number of donor and acceptor hydrogen bonds was zero.

As can be seen in Table 3, 1,8-cineole and sabinene, which have no rotatable single bonds, unfortunately failed to bind to the active binding site of the target protein with hydrogen bonds. The log P for octanol/water was given as 2.417, 2.919, 1.892 and 5.106 values for 1,8-cineole, α-terpinyl acetate, methyleugenol and sabinene, respectively, and was found to be in the range of 95% of drugs (−2.0/6.5). We also calculated the log BB for the brain/blood values of four major constituents, which were determined to be in the range from −3.0/to 1.2. All four compounds indicated very high Caco-2 and MDCK permeability properties (Table 3). Each of these four compounds, which are in compliance with the Lipinski Rule of Five, have a high human oral absorption percentage, have low molecular weights, and are promising as drug candidates; therefore, the possess robust ADME properties, and laurel essential oil may also have such an activity.

## 3. Discussion

Traditional cancer treatment methods such as chemotherapy and radiotherapy have a wide range of side effects, such as cardiotoxicity, myelosuppression, hepatotoxicity and neurotoxicity [28,29,30]. The efforts of modern medicine are directed towards the development of drug delivery systems (DDSs) to increase the effectiveness of these treatments, and to minimize their systemic side effects. The milestone for DDSs is the use of Doxil^®^, which was the first commercially available DDS. Doxorubicin (DOX) should be masked due to its toxic properties to healthy cells. In addition, the therapeutic effect of DOX can be enhanced by encapsulation. Doxil^®^, a DOX-containing liposomal shell, minimizes side effects and increases the efficacy of treatment [31]. In addition to Doxil^®^, Abraxane^®^ [32] and Onivyde^®^ [33] are commercially used anticancer drugs. Nanoparticles of various structures, especially polymeric ones, are the most promising platform for the creation of such DDSs [34,35]. PLGA polymer is one of the most widely used polymers in the development of nanoparticles as anticancer agents [36,37,38,39,40]. Laurel essential oil has been used as an antimicrobial, antioxidant [41,42] diuretic [43], and for the relief of rheumatic pains [44]. Additionally, the essential oil of bay leaves is widely used in the perfume and soap industries [45,46]. However, there are no studies on their anticancer activity in the literature. In this study, we developed PLGA nanoparticles loaded with laurel essential oil for cancer therapy.

The DLS analysis results showed that the mean droplet size of the developed nanoparticles was 211.4 ± 4.031 nm, their PdI value was 0.068 ± 0.016 (Figure 2a), and their zeta potential was −7.87 ± 1.15 mV (Figure 2b).

The PLGA nanoparticle study conducted by Maksimenko et al. reported that the particle size values ranged from 102 to 253 nm, and the zeta potential ranged from −10.1 mV to −11.1 mV [47]. Pereira et al. synthesized PLGA nanoparticles, and they found that the average particle size was 202.5 ± 50.8nm and the PdI was 0.37 ± 0.04 [13]. In a *Cymbopogon citratus* essential oil-loaded PLGA study, the mean particle size, PdI and zeta potential were found to be 277 nm, 0.18, and -16mV, respectively [19]. Average particle size changes between 204 ± 41.3 and 356 ± 54.9 nm, and PdI value changes between 0.25 ± 0.02 and 0.32 ± 0.04 were reported in a clove bud essential oil-loaded nanoparticle assay [48]. 

In another study on PLGA, it was reported that the average particle size was 226.9 nm, the PdI value was 0.004, and the zeta value was −7.41 mV [49]. The average particle size, PdI and zeta potential values obtained in our study are compatible with the previous studies.

Next, the encapsulation efficiency and loading capacity of the developed nanoparticles were calculated. The encapsulation efficiency and loading capacity of synthesized nanoparticles are strategic parameters for the development of effective nanoformulation [50]. The encapsulation efficiency was calculated as 59.25% using Equation (1). In a study conducted with anethole- and carvone-loaded PLGA nanoparticles, it was found that the encapsulation efficiency ranged from 2.12% ± 0.098 to 87.31% ± 5.84% [51]. In another study on PLGA, the encapsulation efficiency was calculated to be 50% [52]. In a study conducted with bergamot essential oil, the encapsulation efficiency was found to be between 28% and 84% [53]. The percentage value we obtained in our study is consistent with the values obtained in the literature [54]. The loading efficiency of the LNEO-loaded PLGA nanoparticles was calculated as 25.65% using Equation (2). In a study conducted with PLGA nanoparticles loaded with carvacrol, the encapsulation efficiency and loading capacity were found to be 26% and 21%, respectively. These results are lower than our results [55]. Compared to the study by Fonte et al., it was found that the encapsulation efficiency is lower and the loading capacity is higher [56].

The in vitro release profile of the LNEO from PLGA nanoparticles showed a biphasic release pattern. The initial rapid release can be explained by the rapid release of the active compound near the surface of the nanoparticles [57,58]. However, the 72-h sustained release may be related to the nature of the LNEO trapped in the core of the PLGA nanoparticles [59]. In general, PLGA degradation is slow. Therefore, the active compound release is also related to the diffusion constant, PLGA swelling and surface PLGA erosion [58].

DNA is the primary pharmacological target of anticancer drugs. Therefore, it is very important to determine the interactions between the new anticancer drug molecule and DNA [25]. Therefore, in this study, we demonstrated the potential of LNEO as a potential anticancer molecule by evaluating its interaction with DNA. The UV-Vis spectra results showed that the interaction of the LNEO with CT-DNA resulted in a 6 nm bathochromic (red) shift and a 93.80% hypochromic effect.

In spectral effects, the vacant π* orbital of the molecule pairs with the π* orbital of the DNA base pairs, resulting in an energy reduction and a reduction of the π-π* transition energy. This is detected by the redshift of the absorption in molecular DNA interaction. At the same time, the empty π* orbital is partially filled with electrons in order to reduce the transition probability, resulting in hypochromism [60,61,62,63]. The hypochromic effect and redshift are typical of the binding of the anticancer molecule to DNA in the intercalation mode [63]. Molecules that intercalatively bind to DNA are used in cancer treatment because they inhibit DNA replication in cancer cells [25,64]. Our results showed that LNEO could be an effective anticancer agent for cancer treatment.

In addition to DNA, PI3K/mTOR signaling pathways, which are accepted as the main regulators for cancer, are also important in the pharmacological targeting of anticancer drugs. PI3Ks are members of the intracellular lipid kinases that regulate the cell metabolism, survival, growth and profile, and mTOR is also a class IV PI3K kinase; both are involved in the regulation of cellular growth and proliferation [26]. Compounds that inhibit both PI3Ka and mTOR, which may offer great potential in cancer therapy, are designed by many companies, including Pfizer, and their PI3K/mTOR dual inhibitory activities are currently being investigated for clinical trials. The antiproliferative activity of laurel essential oil was correlated with interactions on this dual inhibitor of PI3K/mTOR as a receptor [26,27] for molecular docking calculations. The molecular docking results indicated that the amino acid Val 882 in the binding pocket of the PI3K/mTOR receptor, which was chosen in the demonstration of the antiproliferative activity of LNEO, has an important place; the compounds in LNEO interacted with this amino acid residue, and even methyleugenol formed two hydrogen bonds with this residue and achieved the most stable binding to the binding pocket. In addition, the four most abundant compounds also showed hydrophobic interactions with the residues Met 953, Ile 831, Ile 879 and Ile 963, with which the molecule interacts with the target receptor and has antiproliferative activity [64,65]. The ADME results showed that the most abundant compounds in LNEO have low molecular weights (complying with Lipinski’s Rule of Five), have a high percentage of human oral absorption, and ultimately each have promising pharmacokinetic properties to be a drug candidate. Therefore, laurel essential oil also gives such activity.

The interactions of LNEO with DNA and the PI3K/mTOR dual inhibitor indicate that LNEO-loaded PLGA NPs have the potential to be used in cancer therapy as a novel phytotherapeutic agent-based controlled-release system.

## 4. Materials and Methods

### 4.1. Materials

#### Instrumentation and Chemicals

A UV-Vis spectrophotometer (Shimadzu, Kyoto, Japan), a Zeta Sizer Nano ZS (Malvern Instruments, Malvern, UK), a lyophilizer (Biobase, Shandong, China), a centrifuge (Universal 320R, Tuttlingen, Germany), a homogenizer (Bandelin HD, Berlin, Germany) and an Agilent 7890B GC-FID coupled with an Agilent 5977E MS Detector (Santa Clara, CA, USA) were used. PLGA (CAS no. 26780-50-7), polyvinyl alcohol (PVA) (CAS no. 9002-89-5), dichloromethane (DCM) (CAS no. 75-09-2), calf thymus DNA (CT-DNA) and ethidium bromide were purchased from Sigma Aldrich. Tris base, Ethylenediamintetraacetic acid (EDTA), sodium chloride (NaCl), hydrochloric acid (HCl), and sodium hydroxide (NaOH) were purchased from Merck Millipore (Burlington, MA, USA). 

### 4.2. Methods

#### 4.2.1. *Laurus nobilis* Essential Oil

**Figure 12:**
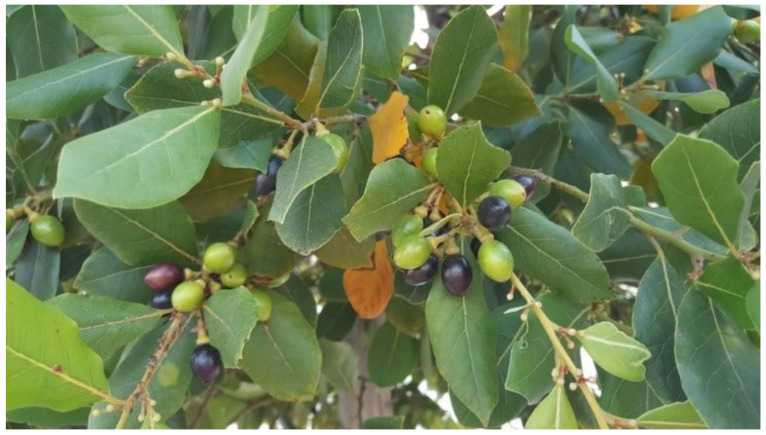
*Laurus nobilis* L. plant.

*Laurus nobilis* L. leaves were collected from the Aegean Agricultural Research Institute (Izmir, Turkey) in June while the flowers were in full bloom, at a latitude and longitude of 38°33′51.8″ N 27°03′01.2″ E with an altitude of 35 m (Figure 12). The plant materials were collected and identified by Prof. Dr. Murat Kartal (Bezmialem Vakıf University, Department of Pharmacognosy, Faculty of Pharmacy), and they were deposited with number “MK15062021 0101” in the Aegean Agricultural Research Institute Herbarium (IZ).

Air-dried *Laurus nobilis* L. leaves (1000 g) were subjected to hydrodistillation using a Clevenger apparatus, as described in the European Pharmacopoeia, for 3 h in triplicate. The essential oils were recovered and dried over anhydrous sodium sulphate. The samples were kept in brown vials at 4 °C until further analysis.

#### 4.2.2. Analysis of the Essential Oil

Solutions of 10% (*v*/*v*) essential oil in n-hexane were subjected to GC-FID/MS analysis. An Agilent 7890B GC-FID (Santa Clara, CA, USA) coupled with an Agilent 5977E electron impact mass spectrometer (Santa Clara, CA, USA) via a two-way capillary splitter was used to identify and quantify the essential oil components. An Agilent G4513A (Santa Clara, CA, USA) auto injector was employed for the injections of 1 µL of the sample solutions. A DB-WAX column (60 m, 0.25 mm, 0.25 µm) was operated with the following temperature program: 70 °C for 15 min, raised to 180 °C at a rate of 2 °C/min. The column temperature was kept isothermal at 180 °C for 5 min, and then increased to 230 °C at a rate of 5 °C/min. Finally, the column temperature was set isothermally at 230 °C for 15 min. The total analysis time was 100 min. Helium was used as a carrier gas with a constant flow of 1.5 mL/min. The split ratio was set to 1:50. The temperatures of the injector port, ion source, quadrupole, MSD transfer line and FID were 250 °C, 230 °C, 150 °C, 250 °C and 220 °C, respectively. The FID air flow was 400 mL/min, and the H_2_ flow was adjusted to 30 mL/min. The mass detector scan range was set to 45–450 *m*/*z*.

The compounds were identified by comparing their spectral data obtained from the Wiley Registry of Mass Spectral Data 9th edition (April 2011) with the NIST 11 Mass Spectral Library (NIST11/2011/EPA/NIH), and by using authentic reference samples. The retention indices were calculated from a co-injected alkane series (C7-C40) compared with previous studies and the NIST online webbook. A quantification was performed by the external standard method, using calibration curves generated by running a GC-FID analysis of representative compounds. All of the analyses were performed in triplicate.

#### 4.2.3. Preparation of the LNEO-NPs

The LNEO-NPs were synthesized using the single-emulsion method [65,66,67,68]. Briefly, 50 mg PLGA was dissolved in 2 mL dichloromethane (DCM). In total, 10 mg LNEO was added to the PLGA solution and mixed on a magnetic stirrer. Then, the LNEO PLGA solution was added dropwise to the 3% PVA solution with an injector. The solution obtained was homogenized by sonication (Bandelin, Sonopuls, Berlin, Germany) for 3 min at 70 W energy in an ice bath. It was stirred on a magnetic stirrer for 16 h in order to remove the solvent from the emulsion. The LNEO-NPs were then purified by applying a three-cycle centrifugation step (at 8000 rpm 40 min). Finally, the LNEO-NPs were filtered through a 0.45 µm cellulose membrane.

#### 4.2.4. Spectrophotometric Analysis of the LNEO

The LNEO analysis was performed using a UV-Vis Spectrophotometer. The maximum absorbance values at 224 nm were obtained for seven different concentrations of the LNEO in ethanol (100, 50, 25, 12.5, 6.25, 3.125, and 1.5625 µg/mL), and the calibration curve of the LNEO was plotted [69]. The calibration curve was used to determine the LNEO’s encapsulation efficiency, loading capacity, and in vitro release.

#### 4.2.5. Average Particle Size, Polydispersity Index (PdI) and Zeta Potential Analyses of the LNEO-NPs

The DLS analyses, including the average particle size, PdI and zeta potential of the LNEO-NPs, were performed using a Zeta Sizer Nano ZS device operating at 25 °C, equipped with a 4.0 mV He-Ne laser (633 nm). The 60 nm and 200 nm polystyrene latex particles were used as references to determine the particle size of the LNEO-NPs. Every sample was freshly prepared. The suspension of the LNEO-NPs was diluted with distilled water, and then measured in triplicate.

#### 4.2.6. Scanning Electron Microscopy (SEM) 

The morphology of the LNEO-NPs was determined using SEM. The LNEO-NPs were dissolved in ultra water by mixing in an ultrasonic bath for 15 min. They were then prepared by dropping 10 µL of the sample onto glass, and were dried at room temperature for 4 h. The SEM images were obtained at 20.00 KX magnification, 10.00 kV electron high tension, and a 15.0-mm working distance with an in-lens detector.

#### 4.2.7. Determination of the Encapsulation Efficiency and Loading Capacity

The encapsulation yield was achieved by the solvent extraction method [19]. Briefly, LNEO-NPs were dissolved in DMSO, vortexed and centrifuged for 15 min (8000). The supernatants were analyzed by UV-Vis spectrophotometry at 224 nm. Empty NPs were used as reference. The total LNEO content was calculated using the curve equation obtained from the standard curve of the LNEO using the absorbance value obtained from the UV-Vis spectrometer. The encapsulation efficiency (EE) was calculated using Equation (1), and the loading capacity (LC) was calculated using Equation (2).

(1) $$\mathrm{EE}\%=\frac{\mathrm{Total}\;\mathrm{amount}\;\mathrm{of}\;\mathrm{loaded}\;\mathrm{LNEO}\;}{\mathrm{Initial}\;\mathrm{amount}\;\mathrm{of}\;\mathrm{the}\;\mathrm{LNEO}\;}\;\times \;100\%$$

(2) $$\mathrm{LC}\%=\frac{\mathrm{Total}\;\mathrm{amount}\;\mathrm{of}\;\mathrm{loaded}\;\mathrm{LNEO}}{\mathrm{NPs}\;\mathrm{after}\;\mathrm{freeze}\;\mathrm{drying}-\mathrm{Weight}\;\mathrm{of}\;\mathrm{the}\;\mathrm{LNEO}}\;\times \;100\%$$

#### 4.2.8. In Vitro Release Profile of the LNEO-NPs

In order to determine the in vitro release profile of the LNEO, 5 mg of the LNEO-NPs were dispersed in distilled water and placed in a dialysis capsule. In total, 75 mL phosphate-buffered saline (60%; pH 7.4) + 40% ethanol were used for the release medium. The samples were incubated at 120 rpm in a horizontal shaking water bath at ambient temperature [69]. The samples were taken at specified time intervals and replaced with an equivalent volume of fresh medium. The samples from the release medium were analyzed using a UV-Vis spectrophotometer. The release amount (%) of the LNEO from the polymeric nanoparticles was calculated using Equation (3).

(3) $$\mathrm{Release}\%=\frac{\mathrm{Released}\;\mathrm{LNEO}}{\mathrm{Total}\;\mathrm{LNEO}}\;\times \;100\%$$

#### 4.2.9. DNA Binding

Calf Thymus DNA (CT-DNA) was used for the determination of the interaction of the LNEO with DNA [25]. The CT-DNA solution in Tris-HCl/NaCl (pH 7.2) buffer gave a UV absorbance ratio of 1.9 at A_260_/A_280_ at 260 and 280 nm wavelengths. This ratio indicates that the CT-DNA does not contain protein [70,71]. The LNEO stock solution was dissolved in ethanol and the required concentration for the experiment, and was prepared by dilution with a buffer. The experiment was performed by keeping the LNEO concentration (40 µg) constant in Tris-HCl/NaCl buffer (pH 7.2) and adding increasing concentrations (0-120 µM) of CT-DNA. After each addition of DNA to the Tris-HCl/NaCl buffer containing LNEO, it was incubated for 5 min, and the absorbance values were recorded.

#### 4.2.10. Molecular Docking and ADME Analysis

In this study, the major components of LNEO from *Laurus nobilis* L. were chosen as potential ligands. As stated in the introduction, laurel leaf and flower essential oils contain 1,8-cineol, α-terpinyl acetate and methyleugenol as their main components [8]. In addition, the leaf essential oil contains at least 48% 1,8 cineole and 70% α-terpinyl acetate [9]. According to the results obtained by GC-MS, the major components of the LNEO from *Laurus nobilis* L. were noted as 62.37% 1,8-cineole, 4.48% α-terpinyl acetate, 0.201% methyleugenol, and 9.76% sabinene. The 1,8-Cineole (PubChem CID2758), α-terpinyl acetate (PubChem CID111037), methyleugenol (PubChem CID7127) and sabinene (PubChem CID18818) molecules were preferred as possible ligands for the molecular docking calculations, and their three-dimensional molecular structures were downloaded from the PubChem site [72], in the light of the data obtained from the analysis results of the essential oil components by GC-MS. Molecular structures downloaded as 3D .sdf files were introduced one by one to the Gaussian package program [73], and were optimized using the DFT/B3LYP-631G(d,p) basis set; the optimized geometries were used for the molecular docking analysis. Using the Lig Prep tool in Maestro version 11.4 in the Schrodinger Software program [74,75,76], the ionization and tautomeric states of the optimized molecular structures were created using the Epic process, and the optimization process was performed using the OPLS force field [77]; possible stereoisomers were produced for each ligand. In the molecular docking analysis, the antiproliferative activity and mechanism of laurel essential oil were supported by their mechanism of action on a dual PI3K/mTOR inhibitor. The phosphatidylinositol 4,5-bisphosphate 3-kinase catalytic subunit gamma isoform with 2.7 Å resolution (PDB 4FA6) was chosen as a potential target for molecular docking from the Protein Data Bank (PDB) [26]. All of the water and ions were deleted, polar hydrogens were added, bond orders were assigned, the charges were defined using PROPKA [78] at pH 7.0, and optimization and minimization processes were applied after preprocess analysis via the Protein Preparation Wizard tool. Using the receptor grid tool, the receptor active site to which the ligands bind was obtained by constructing a grid with a cubic box formed by positioning ligands of certain sizes in the center. After the identification of the atomic groups that could rotate in the binding site of the receptor, ligand–receptor docking was performed. In addition, by using the Qik-Prop module [79,80,81] of the same program, the pharmacokinetic and physicochemical properties of the major chemical compounds in laurel essential oil, such as the molecular weights (M_W_), percent human oral absorption, estimated octanol/water partition coefficient (QPlogPo/w) and Lipinski Rule of Five compliance of each ligand were evaluated.

## 5. Conclusions

In this study, LNEO-NPs were synthesized and characterized. Characterization studies showed that LNEO-NPs have a spherical shape and good distribution in suspension. Moreover, the LNEO-NPs provided the controlled release of LNEO. The usage potential of the LNEO in cancer treatment was investigated using both in vitro and in silico methods.

The antiproliferative activity of the LNEO was achieved by revealing interactions on the dual inhibitor of PI3K/mTOR as a receptor for molecular docking calculations. Based on the strong ADME properties of drug candidate compounds, it may be possible to say that LNEO can be a drug candidate, taking into account the remarkable ADME properties of each of the compounds in the content of laurel essential oil. 

## 6. Recommendations and Future Works

LNEO is an essential oil that has biological activities: namely antioxidant, antimicrobial, and analgesic activities. Although some components of the LNEOs—such as monoterpenes, sesquiterpenes, oxygenated monoterpenes, and phenolic sesquiterpenes—play an important role in cancer treatment, the usage of LNEO is restricted because it is degraded easily by external factors. This restriction can be overcome using controlled release systems. The approach in our present study might be applied to different phytotherapeutic agents. After in vivo applications are conducted, they might become anticancer drug candidates in the future.

## Figures and Tables

**Table 1:** Composition of *Laurus nobilis* leaf essential oil.

No	Compound	R.T (Min)	R.I_L_	R.I_C_	Amount (%)
1	*α*-Pinene	7.582	1035	1037	5.995 ± 0.0157
2	Camphene	8.864	1065	1068	0.928 ± 0.0086
3	*β*-Myrcene	10.406	1118	1120	1.127 ± 0.0043
4	Sabinene	10.898	1125	1129	9.767 ± 0.0129
5	*β*-Pinene	12.871	1167	1165	4.685 ± 0.0102
6	Limonene	15.454	1208	1206	1.395 ± 0.0021
7	1.8-Cineole	16.525	1212	1218	62.370 ± 0.0162
8	*p*-Cymene	20.690	1280	1274	0.705 ± 0.0035
9	Linalool	40.983	1543	1549	4.386 ± 0.0078
10	Terpinen-4-ol	44.368	1601	1608	0.928 ± 0.0041
11	*α*-Terpinyl Acetate	49.992	1687	1690	4.485 ± 0.0017
12	*α*-Terpineol	50.061	1694	1693	0.137 ± 0018
13	Methyleugenol	66.680	2033	2036	0.201 ± 0.0021
Total Identified					94.411
Monoterpenes (1–6, and 8) *					22.969
Oxygenated Monoterpenes (7, 9–12) *					71.241
Phenylpropanoids (13) *					0.201

* These numbers refer to the components. R.T: Retention time (min). R.I_L_: Retention indices derived from the literature and NIST webbook database. R.I_C_: Calculated retention indices.

**Table 2:** The docking score energies and probable interactions of major ingredients of LNEO from *Laurus nobilis* L. with receptor as a potent inhibitor of PI3Ka and mTOR (PDB code: 4FA6).

Ligands	1,8-Cineole	α-Terpinyl Acetate	Methyleugenol	Sabinene
Docking Score (Kcal/mol)	−4.89	−4.68	−5.97	−5.26
H-bonding Interaction (Angstrom)	-	Asp 950 (2.58)	Val 882(2.30) Val 882(2.43)	-
Salt Bridge Interaction	-	-	-	-
Cation-π Interaction	-	-	-	-
Hydrophobic Residues	Val 882, Ile 881, Ile 879, Trp 812, Pro 810, Met 804, Leu 838, Phe 832, Ile 831, Leu 838, Met 953, Phe 961, Ile 963, Phe 965, Tyr 867, Cys 869	Val 882, Ile 881, Ile 879, Ala 885, Ala 889, Met 953, Ile 952, Tyr 867, Phe 961, Ile 963, Ile 831, Met 804, Ala 805, Trp 812, Pro 810	Val 882, Ala 885, Met 804, Phe 965, Ile 963, Phe 961, Ile 879, Ile 881, Leu 865, Pro 866, Tyr 867, Leu 845, Ile 831, Leu 838, Pro 810, Trp 812, Met 953	Val 882, Ile 881, Ile 879,Ala 885, Trp 812, Pro 810, Met 804, Phe 832, Ile 831, Phe 965, Ile 963, Phe 961, Leu 845, Met953, Tyr 867, Cys 869
Polar Residues	His 962, Thr887, Asn 951, Ser 806	Asn 946, Asn 951, Thr 887, Ser 806	His 962, Ser 806, Asn 951, Thr 887, Thr 886	Thr 887, Ser 806, His 962, Asn 951
Charged (positive) Residues	Arg 947, Lys 890, Lys 833, Lys 808	Lys 833, Lys 802, Lys 890	Lys 883, Lys 890, Lys 833	Lys 833, Lys 890, Lys 808
Charged (negative) Residues	Asp 950, Asp 964, Ash 841, Asp 836, Glu 880	Asp 950, Glu 880, Asp 964	Asp 964, Glu 880, Ash8 41, Asp 884	Asp 950, Asp 964, Glu 880
Glycine	-		Gly 868	Gly868

**Table 3:** The calculated ADME properties of the major components of the LNEO from *Laurus nobilis* L. docked with a dual PI3K/mTOR inhibitor.

Major Components of the LNEO	1,8-Cineole	α-Terpinyl Acetate	MethyleuGenol	Sabinene	
Docking Score (kcal/mol)	−4.89	−4.68	−5.97	−5.26	
**Principal Descriptors**					**(Range 95% of Drugs)**
Solute Molecular Weight	154.252	200.320	186.294	138.252	(130.0/725.0)
Solute Dipole Moment (D)	1.624	2.112	2.633	0.160	(1.0/12.5)
Solute Total SASA	373.729	458.423	448.882	381.877	(300.0/1000.0)
Solute Hydrophobic SASA	373.729	412.688	448.882	381.877	(0.0/750.0)
Solute Hydrophilic SASA	0.000	45.735	0.000	0.000	(7.0/330.0)
Solute Carbon Pi SASA	0.000	0.000	0.000	0.000	(0.0/450.0)
Solute Weakly Polar SASA	0.000	0.000	0.000	0.000	(0.0/175.0)
Solute Molecular Volume (Å^3^)	618.965	796.485	752.599	626.954	(500.0/2000.0)
Solute vdW Polar SA (PSA)	7.264	28.091	17.239	0.000	(7.0/200.0)
Solute No, of Rotatable Bonds	0.000	4.000	4.000	1.000	(0.0/15.0)
Solute as Donor—Hydrogen Bonds	0.000	1.000	0.000	0.000	(0.0/6.0)
Solute as Acceptor—Hydrogen Bonds	0.750	1.000	3.400	0.000	(2.0/20.0)
Solute Globularity (Sphere = 1)	0.940	0.906	0.891	0.928	(0.75/0.95)
Solute Ionization Potential (eV)	10.363	10.705	10.465	10.610	(7.9/10.5)
Solute Electron Affinity (eV)	−2.516	−2.449	−2.441	−2.806	(−0.9/1.7)
**Predictions for Properties:**					
QP Polarizability (Å^3^)	18.617	23.153	21.397	18.295	(13.0/70.0)
QP log P for hexadecane/gas	4.348	6.379	5.285	4.311	(4.0/18.0)
QP log P for octanol/gas	5.449	9.483	7.702	4.817	(8.0/35.0)
QP log P for water/gas	1.357	4.129	3.266	−1.113	(4.0/45.0)
QP log P for octanol/water	2.417	2.919	1.892	5.106	(−2.0/6.5)
QP log S for aqueous solubility	−2.985	−3.103	−2.730	−5.202	(−6.5/0.5)
P log S—conformation independent	−3.615	−2.256	−2.730	−5.202	(−6.5/0.5)
QP log K HSA Serum Protein Binding	0.214	0.152	−0.277	0.396	(−1.5/1.5)
QP log BB for brain/blood	0.597	−0.049	−0.464	0.957	(−3.0/1.2)
No, of Primary Metabolites	1	0	0	0	(1.0/8.0)
Predicted CNS Activity (-- to ++)	++	+/−	+/−	++	
HERG K+ Channel Blockage: log IC_50_	−2.506M	−3.046	−3.289	−2.702M	(concern below −5)
Apparent Caco-2 Permeability (nm/sec)	9906	3649	9906	9906	(<25 poor, >500 great)
Apparent MDCK Permeability (nm/sec)	5899	2004	5899	5899	(<25 poor, >500 great)
QP log Kp for skin permeability	−0.923	−1.978	−1.331	1.079	(Kp in cm/hr)
Jm, max transdermal transport rate	4.476	1.661	16.199	10.415	(micrograms/cm^2^-hr)
Lipinski Rule of 5 Violations	0	0	0	1	(maximum is 4)
Jorgensen Rule of 3 Violations	0	0	0	0	(maximum is 3)
% Human Oral Absorption in GI (±0%)	100	100	100	100	(<25% is poor)
Qual, Model for Human Oral Absorption	HIGH	HIGH	HIGH	HIGH	(>80% is high)

## Data Availability

Not applicable.

## Abbreviations

LNEO	*Laurus nobilis* L. essential oil
PLGA	poly lactic-co-glycolic acid
NPs	nanoparticles
LNEO-NPs	*Laurus nobilis* L. essential oil loaded poly lactic-co-glycolic acid (PLGA) nanoparticles
GC-MS	gas chromatography-mass spectrometer
UV-Vis	ultraviolet–visible
DLS	dynamic light scattering
PdI	polydispersity index
PBS	phosphate-buffered saline
SD	standard deviation
SEM	scanning electron microscopy
CT-DNA	calf thymus DNA
PI3K/mTOR	phosphatidylinositol 3-kinase/mammalian rapamycin target
ADME	absorption, distribution, metabolism, excretion
